# Retrosplenial and Hippocampal Synchrony during Retrieval of Old Memories in Macaques

**DOI:** 10.1523/JNEUROSCI.0001-22.2022

**Published:** 2022-10-19

**Authors:** Ahmed T. Hussin, Saman Abbaspoor, Kari L. Hoffman

**Affiliations:** ^1^Department of Biology, Centre for Vision Research, York University, Toronto Ontario M3J 1P3, Canada; ^2^Departments of Psychology; ^3^Biomedical Engineering, Vanderbilt Vision Research Center, Vanderbilt Brain Institute, Vanderbilt University, Nashville, Tennessee 37240

**Keywords:** alpha oscillation, episodic memory, memory consolidation, remote memory, theta oscillation, visual search

## Abstract

Memory for events from the distant past relies on multiple brain regions, but little is known about the underlying neural dynamics that give rise to such abilities. We recorded neural activity in the hippocampus and retrosplenial cortex of two female rhesus macaques as they visually selected targets in year-old and newly acquired object-scene associations. Whereas hippocampal activity was unchanging with memory age, the retrosplenial cortex responded with greater magnitude alpha oscillations (10–15 Hz) and greater phase locking to memory-guided eye movements during retrieval of old events. A similar old-memory enhancement was observed in the anterior cingulate cortex but in a beta2/gamma band (28–35 Hz). In contrast, remote retrieval was associated with decreased gamma-band synchrony between the hippocampus and each neocortical area. The increasing retrosplenial alpha oscillation and decreasing hippocampocortical synchrony with memory age may signify a shift in frank memory allocation or, alternatively, changes in selection among distributed memory representations in the primate brain.

**SIGNIFICANCE STATEMENT** Memory depends on multiple brain regions, whose involvement is thought to change with time. Here, we recorded neuronal population activity from the hippocampus and retrosplenial cortex as nonhuman primates searched for objects embedded in scenes. These memoranda were either newly presented or a year old. Remembering old material drove stronger oscillations in the retrosplenial cortex and led to a greater locking of neural activity to search movements. Remembering new material revealed stronger oscillatory synchrony between the hippocampus and retrosplenial cortex. These results suggest that with age, memories may come to rely more exclusively on neocortical oscillations for retrieval and search guidance and less on long-range coupling with the hippocampus.

## Introduction

**Figure 1.**
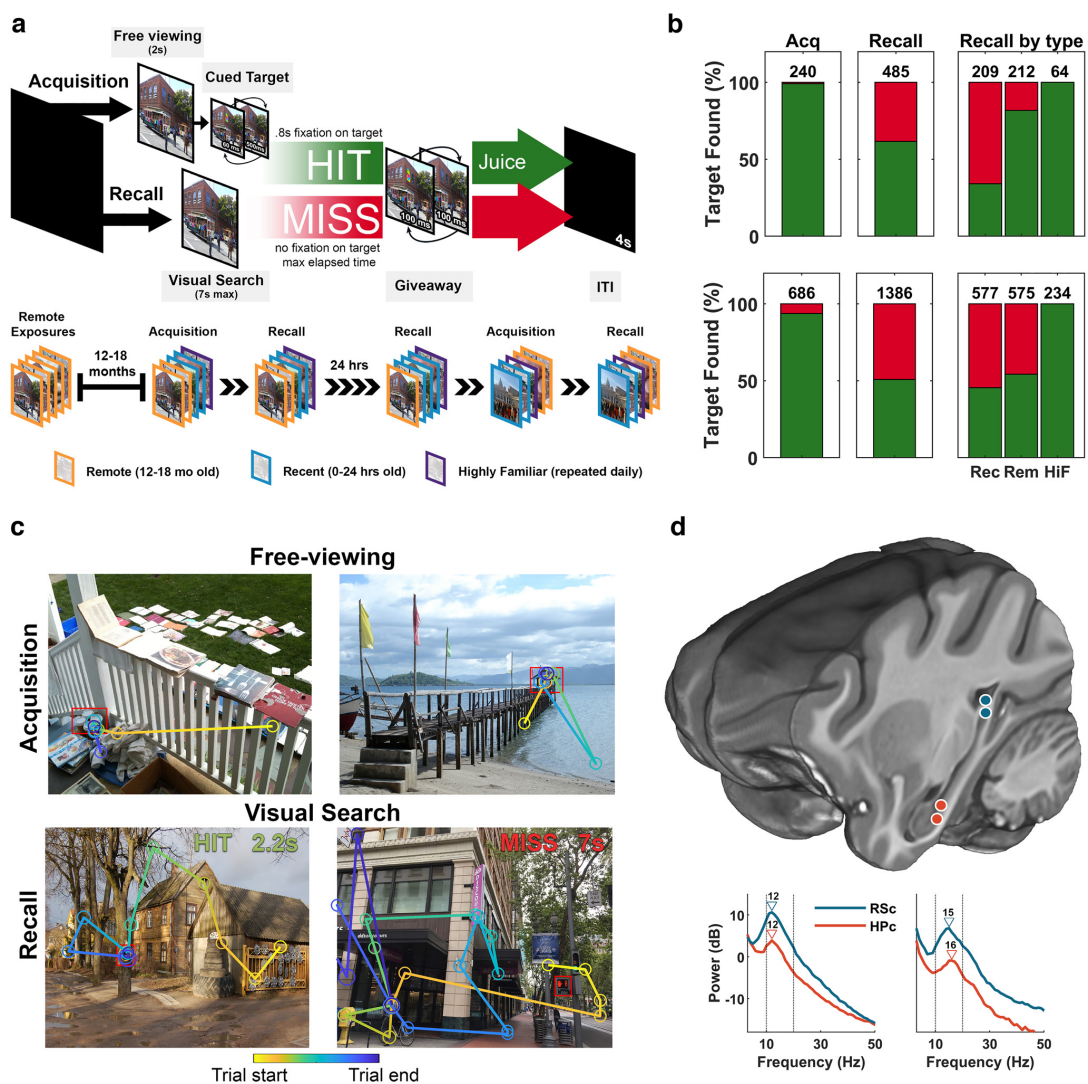
Experimental design and task performance. ***a***, Top, Acquisition trials begin with 2 s of free viewing followed by target cuing. During recall, the scene is presented without this cue, requiring the target location to be remembered. The trial ends when gaze is maintained within the target area for 0.8 s, for which a fluid reward is delivered (HIT), or when the maximum trial time of 7 s is reached (MISS). A giveaway presents the cued target for a longer duration (100 ms) at the end of a trial followed by an intertrial interval of 4 s. Bottom, Sets were composed of three stimulus types consisting of remote scenes, which had been presented 12–18 months prior; recent scenes, which were novel scenes at the start of these sessions; and six highly familiar scenes, which were presented repeatedly throughout the study. Equal numbers of remote and recent scenes were presented in each set, randomly interleaved. Recall sets were shown after the acquisition set on the same day and 1 d following acquisition but before a new acquisition set. ***b***, Top from left, HIT rate for acquisition (Acq) trials, recall trials, and recall by scene type for monkey RI. Bottom, Same as top but for monkey LE. Values above bars indicate number of HITS in respective conditions. Rec, Recent; Rem, remote; HiF, highly familiar. ***c***, Top, Example scan paths during free viewing on remote acquisition trials. The target is outlined in red. Directed gaze toward the (uncued) target indicates memory for the target. Bottom, Example scan paths for a HIT (remembered trial) and a MISS (forgotten trial) during recall trials. Top right, Search time in seconds. ***d***, Top, Imaging-localized electrode positions, blue, RSC; red, HPC. Bottom, Mean power during search for RSC (blue) and HPC (red; left, RI; right, LE). Vertical lines indicate 10 and 20 Hz; triangles indicate peak frequency.

The ability to recall events that transpired months or years in the past is a crowning achievement of primate cognition and one that is fundamental to our daily lives. Both the hippocampus (HPC) and retrosplenial cortex (RSC) are important structures for forming long-lasting memories of spatiotemporally distinct events (Maguire, 2001; Spreng et al., 2009; Vann et al., 2009; Aggleton, 2010; Dickerson and Eichenbaum, 2010; Smith et al., 2012; Katche et al., 2013; Miller et al., 2014; Ritchey et al., 2015; Todd and Bucci, 2015; Chrastil, 2018; Rolls and Wirth, 2018). The neural mechanisms within these structures that give rise to retrieval over large time spans is unclear, although the retrosplenial cortex may assume a greater, more independent role over time (Vann et al., 2009; Miller et al., 2014; Mao et al., 2018; de Sousa et al., 2019; Milczarek and Vann, 2020; Opalka and Wang, 2020). Tests of this role have been limited by (1) species-adapted tasks in rodents that use shorter delays and restricted memoranda, (2) a historic emphasis on hippocampal physiology, and (3) indirect access to neuronal populations in humans. Here, we measure the neural activity in retrosplenial cortex and the hippocampus of macaques as they perform episodic-like memory tasks on unique item-in-visuospatial scenes (Chau et al., 2011). Some of these stimuli had not been seen for more than a year, whereas other stimuli were presented for the first time and recalled within the same session or within 24 h (Fig. 1*a*). We asked whether the HPC or RSC would show changes in neural population activity as a function of retrieval of events learned 1–1.5 years earlier compared with newly learned events.

## Materials and Methods

### Surgical procedures

In a sterile surgical procedure, two adult female macaques (*Macaca mulatta*, LE and RI, ∼12 and 10 kg, respectively) were implanted with 12 indwelling flexible, polyamide-based intracortical 16-channel linear electrode arrays with contacts at 130 µm spacing (prototypes of MicroFlex electrodes, Blackrock), targeting the hippocampus, anterior cingulate cortex (ACC), and retrosplenial cortex as described in Talakoub et al. (2019). All surgical and experimental protocols were conducted with approval from the local ethics and animal care authorities (Animal Care Committee, Canadian Council on Animal Care). Surgery was performed, and data were collected at York University, Toronto, Canada.

### Experimental design and statistical analysis

Both monkeys completed a memory-guided visual search task 12–18 months before the present recordings. During this task, a target object was embedded in a naturalistic scene and presented alongside other objects in scenes, which made up the remote stimuli used in this study. During the present experiments, the animals performed two trial types within each daily session: acquisition and recall trials. During an acquisition trial, the scene is displayed for 2 s and the animal is allowed to view the scene freely, followed by presentation of a target unique to the scene that is cued by alternating between original (500 ms) and complementary colors (60 ms), making the target location salient and appearing to pop out to the observer. Target cuing began at 2 s and continued until the target was selected (designated as HIT) or until 7 s had passed (designated as MISS). Selection of the target was accomplished by holding the gaze in the target region for a prolonged duration (≥800 ms). Once the target was cued, the task became trivially easy for the macaques. In contrast, during a recall trial, the scene is presented without the cue, and the animal had 7 s to find and select the (uncued) target for juice reward (HIT or remembered), or the trial ended without reward (MISS or forgotten). All trials ended with a giveaway, where the original and color-modified scenes alternate (100 ms each × 5) revealing the target to the animal. An intertrial interval of 4 s of black screen followed each trial (Fig. 1*a*).

Scenes were grouped into sets of 12 (monkey RI) or 16 (monkey LE) scenes. The number of scenes in a set was designed to account for individual performance differences. Each set had three types of scenes, namely, recent, remote, and highly familiar. Recent scenes were novel to the animal during the first acquisition presentation. Remote scenes were scenes used during initial task training 12–18 months prior. Highly familiar scenes were a preselected subset of six remote scenes that were repeated regularly throughout the experiment (two were included in each set) and therefore have a high HIT rate. There was an insufficient number of these highly familiar trials for analysis, and therefore they were not considered further in this study. Presentation order of scene types was randomized within the set. Recall sets immediately followed the acquisition sets within a daily session. In addition, the session on the following day began with the recall sets from the previous day. Two new sets were presented each day (i.e., 24 and 32 new scenes per day, for the two animals, respectively). Daily sessions started and ended with a 5 min rest period where a black screen was presented. Eye movements were recorded at 1250 Hz using video-based eye tracking (IViewX Hi-Speed Primate Remote Infrared Eye Tracker). For the analysis we excluded trials where the animals spent >20% of trial duration looking off screen (monkey LE, 238/2755 trials or 8%; monkey RI, 50/1310 trials or 4%) to ensure that only trials where the animals were attending to the task were included.

Proportions of hit rate and bout occurrence across scene types were compared using a two-tailed chi-square test for comparing proportions. Search times were compared using a Kruskal–Wallis test. Time-frequency spectra were compared using nonparametric permutation tests using the Monte Carlo sampling method and a cluster-based correction for multiple comparisons. To test for statistical significance of differences between phase concentration and synchrony [weighted phase lag index (wPLI) values] during the recent and remote conditions, we performed a nonparametric permutation test with the difference in phase concentration or coherence between conditions as our test statistic. The test statistic was calculated for each frequency bin, then bins whose statistic value was <2.5th or >97.5th percentile were selected, and cluster-level statistics were calculated by summing the test statistic within a cluster. This testing method corresponds to a two-tailed test with a false-positive rate of 5% corrected for multiple comparisons across frequencies (Nichols and Holmes, 2002; Maris and Oostenveld, 2007; Turesson et al., 2012).

### Neural recordings

Local field potentials (LFP) were recorded simultaneously from the hippocampus and anterior cingulate and retrosplenial cortices, digitally sampled at 32 kHz using a Digital Lynx acquisition system (Neuralynx) and filtered between 0.5 Hz and 2 kHz. Because of problems with the implant connection to the ACC contacts of one animal, results from ACC were evaluated for only the remaining animal (RI). No well-isolated units were recorded with these arrays, so all analysis is based on local field potentials. Inclusion criteria for any probe consisted of CT/MR coregistration or postexplant MR verification of location within a region of interest, including visualization of marker lesions, for the subset of probes in which they had been delivered. Along the 16-channel probe, occasional channels were excluded based on the signal not following biological 1/f spectra or otherwise not showing signal. The remaining neural signals were downsampled to 1 kHz, and a notch filter (59.9–60.1 Hz) was used to remove 60 Hz noise. All off-line behavioral and neural analysis was conducted in MATLAB using custom-written scripts and in FieldTrip, https://www.fieldtriptoolbox.org/).

### Generalized eigendecomposition

We implemented generalized eigendecomposition (GED) for source separation of the LFP in the multichannel arrays (based on methodology described in Cohen, 2018) because of nonorthogonal alignment of the probes to laminae and our inclusion of multiple probes within an area. Analyses of power, phase concentration, and phase synchrony involved the use of linear spatial filters to provide a weighted combination of electrode activity, isolating sources of independent variance in multichannel data. The spatial filters were defined by the GED of covariance matrices of the channels. The two separate covariance matrices selected result in eigenvectors that maximally differentiate them. If the signal features to be accentuated and those to be attenuated are designated by S and R, respectively, the eigendecomposition problem can be written as SW = WRΛ. The solution to this problem yields W, which is a matrix of eigenvectors, and Λ, which is a diagonal matrix of eigenvalues. The resultant filters, defined by eigenvectors, are then applied to the multichannel electrode time series to obtain a set of component time series. If GED was unable to differentiate between various sources of variance, shrinkage regularization was used at 1%.

For power and phase concentration analyses, the S matrix was created from 1 s of signal after scene onset (start of trial) and the R matrix from 1 s of baseline activity before the scene onset. In this design, we sought to attenuate continuous noise in the signal and accentuate the dynamics and signal sources producing those dynamics that are relevant to the task. For phase synchrony analysis, we created the S matrix from the bandpass-filtered electrode time series in 10–20 Hz. The R matrix was then formed from the broadband electrode time series. In this case, the column in W with the highest corresponding eigenvalue then corresponds to the eigenvector that maximally enhances the 10–20 Hz frequency activity. The inputs to the GED were signal from multiple channels and trials from a given probe, and the output was a single weighted time series component per trial per probe. The analyses that follow use the primary resultant GED component that represents the weighted combination of activity from multiple channels in each probe. This source isolation method circumvents the trade-off of excluding simultaneously recorded channels arbitrarily, when they may contribute informative signal, while ensuring that the dependence of signals across channels is not falsely considered as independent samples of the time series. Qualitatively, use of single-channel LFP yielded very similar results, although it suffers from the aforementioned arbitrary exclusion issue.

### Spectral analysis

Grand power was computed using a Fourier transform and a Hanning multitaper frequency transformation, averaging over the whole duration of search trials including both acquisition and recall trials (*N* trials, LE, 1152; RI, 422). Mean power spectral density was examined in 500 ms windows with a 1 ms sliding window conducted on individual trials then averaged across trials. For mean time-frequency spectra, we implemented a Morlet wavelets multitaper transformation with a width of five cycles and a frequency step size of 1 Hz.

### Phase concentration

To examine phase alignment with eye movement we inspected the LFP signal in 600 ms windows centered around fixation onsets (perifixation signal). We examined all recall trial fixations split by remembered and forgotten trials. We bandpass filtered the perifixation neural signal between 4 and 9 Hz, which captures the main components of the saccade-evoked LFP response (see Fig. 4). This allows us to quantify the consistency of the main response time course and to directly compare with the previous results from hippocampal LFP (Hoffman et al., 2013; Jutras et al., 2013; Katz et al., 2020; Kragel et al., 2020). To accomplish this, we used the phase angles of the Hilbert transform to compute the mean resultant vector length (or phase concentration). Circular statistical analyses were performed using the Circular Statistics Toolbox for MATLAB (Berens, 2009).

### Bout detection

For detection of oscillatory bouts of activity in the dominant frequency band, the RSC signal from all trials was bandpass filtered between 10 and 15 Hz. Bouts were defined as time periods in which the signal exceeded a threshold of 2 SDs above the envelope mean and for a minimum duration of 100 ms above 1 SD. Bout amplitude was defined as the maximum of the envelope within a bout. Control bouts were chosen under the same criteria but in the opposite direction (i.e., −2 SDs) to identify windows of time with the weakest RSC 10–15 Hz power.

### Phase synchrony

Interarea phase synchrony during bouts was estimated from the cross-spectral density of the RSC signal and the corresponding HPC signal using the debiased wPLI. The debiased wPLI measure of phase synchronization minimizes the influence of volume conduction, noise, and the sample-size bias (Vinck et al., 2011), allowing us a more conservative measure of synchrony in that it reduces our type 1 error, while recognizing that zero-lag effects may go undetected (type II error).

## Results

We recorded 62 sessions from two macaques (LE, 37; RI, 25), both of which had a >90% hit rate on acquisition trials (Fig. 1*c*, example scan paths during search). During recall, they had a higher hit rate for old (remote) compared with newly acquired (recent) scenes [Fig. 1*b*; LE, χ^2^(1,395) = 35, *p* < 0.01; RI, χ^2^(1,395) = 44, *p* < 0.01], indicating memory savings of remotely learned scene targets. Correspondingly, average search times were faster for remote scenes than for recent scenes (remote, recent trials, respectively; RI, 3.20 s, 5.91 s; LE, 5.88 s, 6.56 s; H[1] = 99.22, *p* = 2.25e-23). Considering only remembered recall trials, average search times were 2.80 s for animal LE (SD = 1.78 s) and 2.11 s for animal RI (SD = 1.64 s).

**Figure 2.**
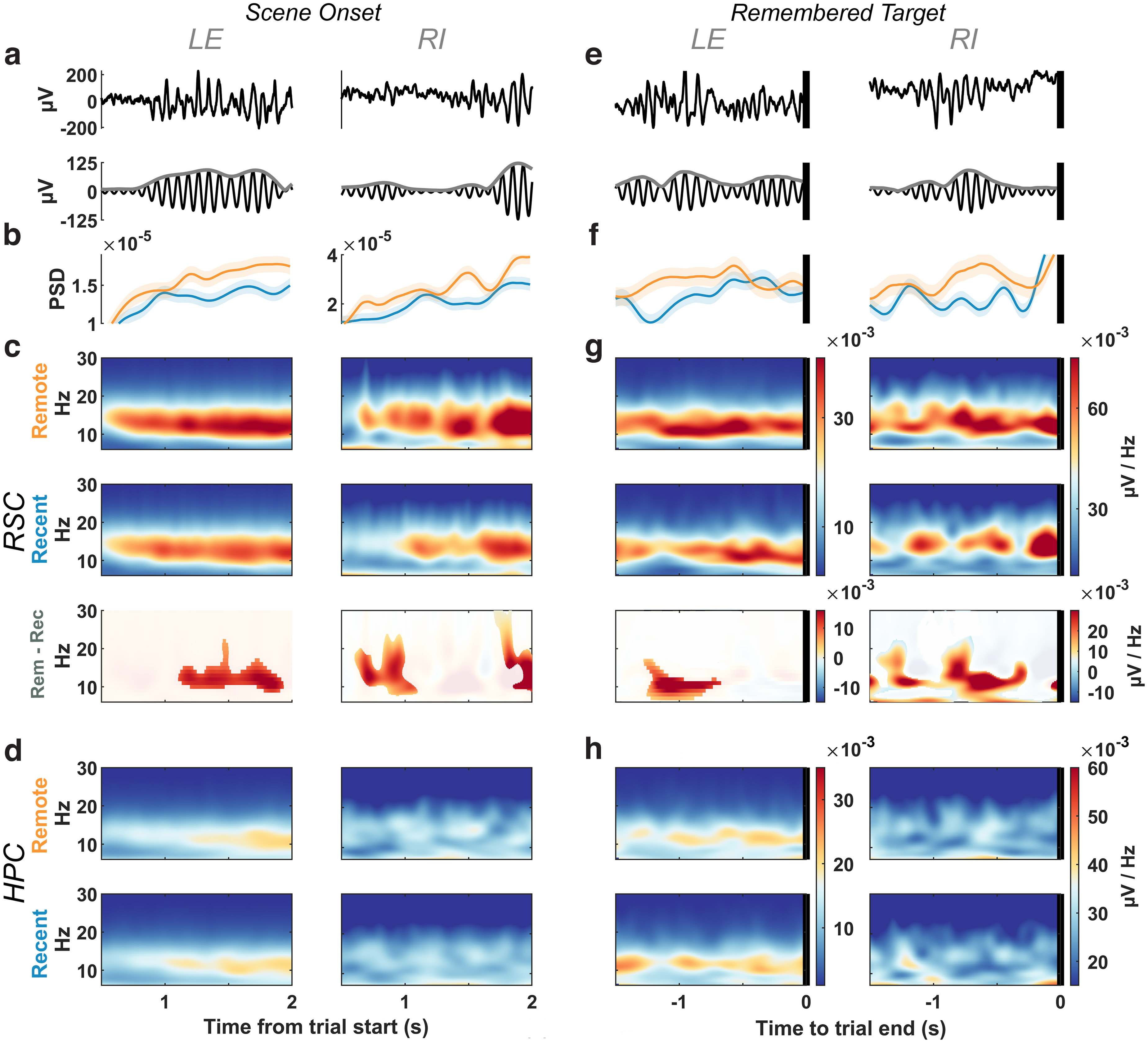
Greater alpha power (10–15 Hz) during remote than recent scene recall in the RSC but not the HPC. ***a***, Top, Example broadband signal from RSC during the first 2 s after scene onset. Bottom, From top example, 10–15 Hz filtered signal. LE and RI indicate the study animals. Abscissas as in ***d***. ***b***, Mean power spectral density of 10–15 Hz band using 500 ms windows in 1 ms steps. Shading indicates 95% bootstrap confidence intervals. ***c***, Top, Mean spectrogram of remote trials (LE, *n* = 594; RI, *n* = 152); middle, for recent trials (LE, *n* = 605; RI, *n* = 240); bottom, remote-to-recent difference spectrogram with the nonmasked region representing areas with a difference of *p* < 0.05 in a cluster-based permutation test corrected for multiple comparisons. ***d***, Top, Mean spectrogram of remote trials in the hippocampus; bottom, recent. ***e–h***, Same as ***a–d***, respectively, but aligned to the last 1.5 s of remembered trials.

**Figure 3.**
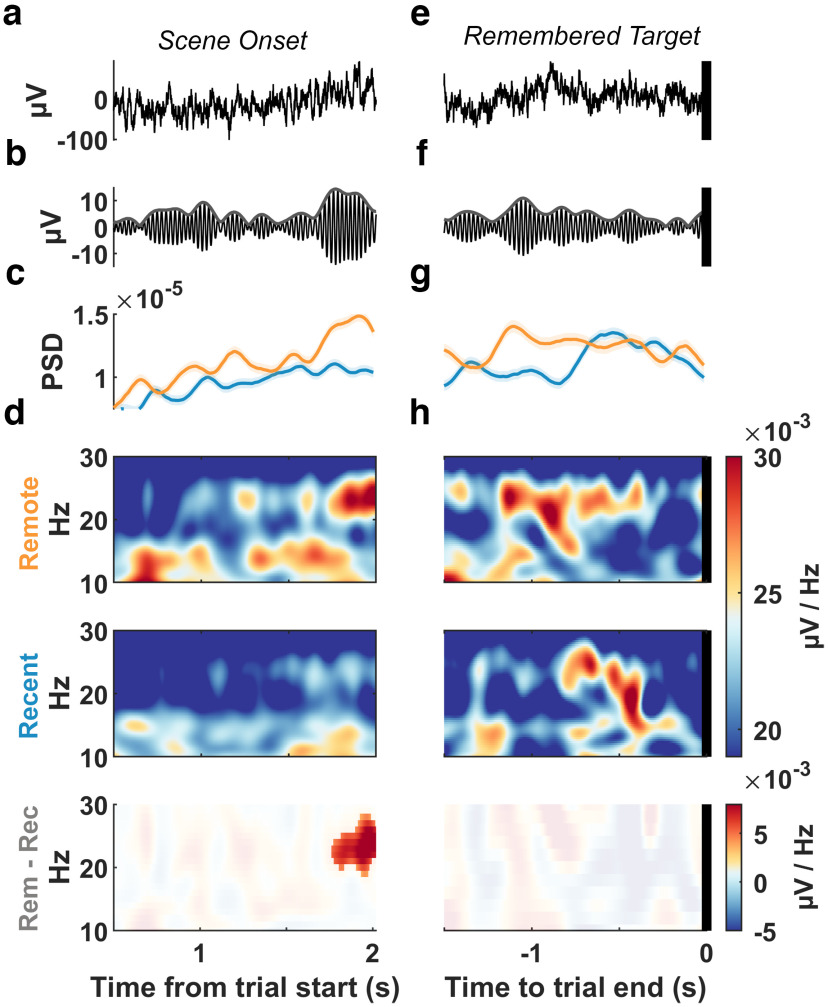
ACC exhibits greater 21–26 Hz power during remote scenes. ***a***, Example broadband signal during the first 2 s after trial onset when a scene is presented (power spectrum not shown here showed a peak of ∼23 Hz). ***b***, The 21–26 Hz filtered signal. ***c***, Mean power spectral density in the 21–26 Hz band using 300 ms windows in 1 ms steps. Shading indicates 95% bootstraped confidence intervals. ***d***, Top, Mean spectrogram of remote trials (*n* = 289); middle, recent trials (*n* = 193); bottom, remote-to-recent difference spectrogram with the nongrayed region representing clusters with a difference of *p* < 0.05 in a permutation test. ***e–h***, Same as ***a–d*** but for the trial end epoch of remembered trials (remote, *n* = 45; recent, *n* = 65).

Using multichannel recordings from 12 indwelling, 16-channel linear electrodes (Talakoub et al., 2019), we obtained local field potentials from several structures, including the RSC and the HPC (Fig. 1*d*). The spectral power during visual search showed a prominent narrow-band peak between 10 and 20 Hz in the RSC and HPC of both animals (Fig. 1*d*). When we aligned the RSC signals to scene onset and remembered-target selection consisting of the last 1.5 s before target selection on remembered trials, we found greater RSC power as early as 0.5 s after scene onset (Fig. 2*b*,*c*) and in the final second before target selection on remembered trials (Fig. 2*f*,*g*) using multiple-comparison corrected permutation tests. We found no consistent differences in HPC power between remote and recent trials (Fig. 2*d*,*h*) at any frequencies from 3 to 80 Hz. The ACC was recorded in one animal, showing oscillations in a higher 23 Hz frequency band that was stronger for remote than for recent trials, but only during scene onset (Fig. 3).

We used general linear models (GLM) to quantify whether event age predicts magnitude of the alpha (10–15 Hz) RSC oscillation during acquisition trials while accounting for other predictors, including the target location on the screen and animal. We modeled acquisition trials to determine whether alpha power differed between learning (recent scenes) and retrieval (remote scenes). RSC alpha power was predicted by memory age (remote greater than recent) and animal (RI greater than LE) but not target location (*F*_(3,545)_ = 49.58, *p* = 2.2 × 10^−16^, adjusted *R*^2^ = 0.21; scene age *t* = −3.08, *p* < 0.01; animal *t* = 11.92, *p* < 0.001). Similarly, conditioning alpha power on the recall trial type, we found that memory age (remote), and animal (RI) were significant predictors (*F*_(5,984)_ = 22.31, *p* = 2.2 × 10^−16^, adjusted *R*^2^ = 0.097; scene age, *t* = −4.07, *p* < 0.001; animal, *t* = 10.14, *p* < 0.0001; see above, Materials and Methods for GLM details).

**Figure 4.**
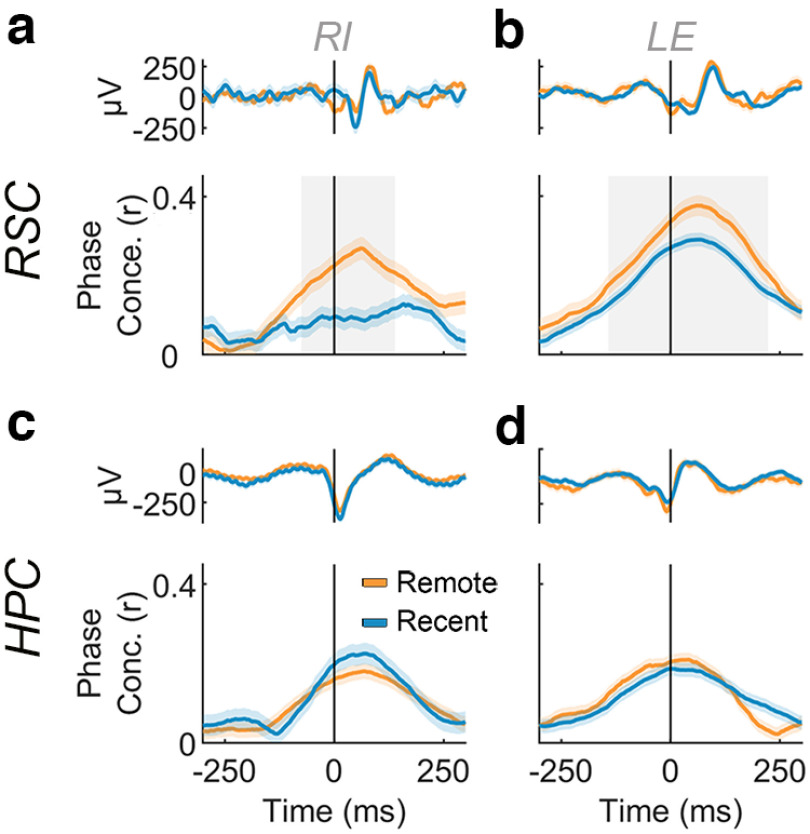
Phase concentration of RSC LFPs aligns to visual fixations during remembered scenes. ***a***, Top, Mean LFP locked to fixations on remembered trials. Bottom, Mean phase concentration of RSC around the time of fixations for monkey RI (remote, *n* = 2134; recent, *n* = 1042). Remote trials are depicted in orange and recent trials in blue. Light shading around mean traces represents 95% bootstrapped confidence intervals. Gray shading represents *p* < 0.05 difference between remote and recent phase concentrations in a two-tailed cluster-based permutation test. Abscissa as in ***c***. ***b***, Same as ***a*** but for animal LE (remote, *n* = 1267; recent, *n* = 2921). ***c***, ***d***, Similar to ***a*** and ***b***, respectively, but for the hippocampus of each animal (LE remote, *n* = 2054; recent, *n* = 1031; RI remote, *n* = 1297; recent, *n* = 2972).

If memory-related retrosplenial cortex activity guides visual search during retrieval, one would expect greater coupling to the eye movements that underlie a successful search, similar to previous observations with hippocampal signals (Hoffman et al., 2013; Jutras et al., 2013). Specifically, we measured phase alignment of the LFP in both RSC and HPC to eye movements during successful retrieval, evaluated separately for remote and recent trials. Phase alignment was greater in the RSC (Fig. 4*a*,*b*) on remote compared with recent scenes, beginning shortly before fixation onset (LE, −75 ms; RI, −175 ms) and lasting until 125–200 ms postfixation. This effect was limited to remembered trials. In contrast to the RSC, the HPC phase alignment did not vary by scene age (Fig. 4*c*,*d*). Thus, on a general background of phase locking to visual fixations across regions, the retrosplenial cortex alone showed greater coupling to search during old memory retrieval, consistent with a role for this region in memory-based guidance of actions.

**Figure 5.**
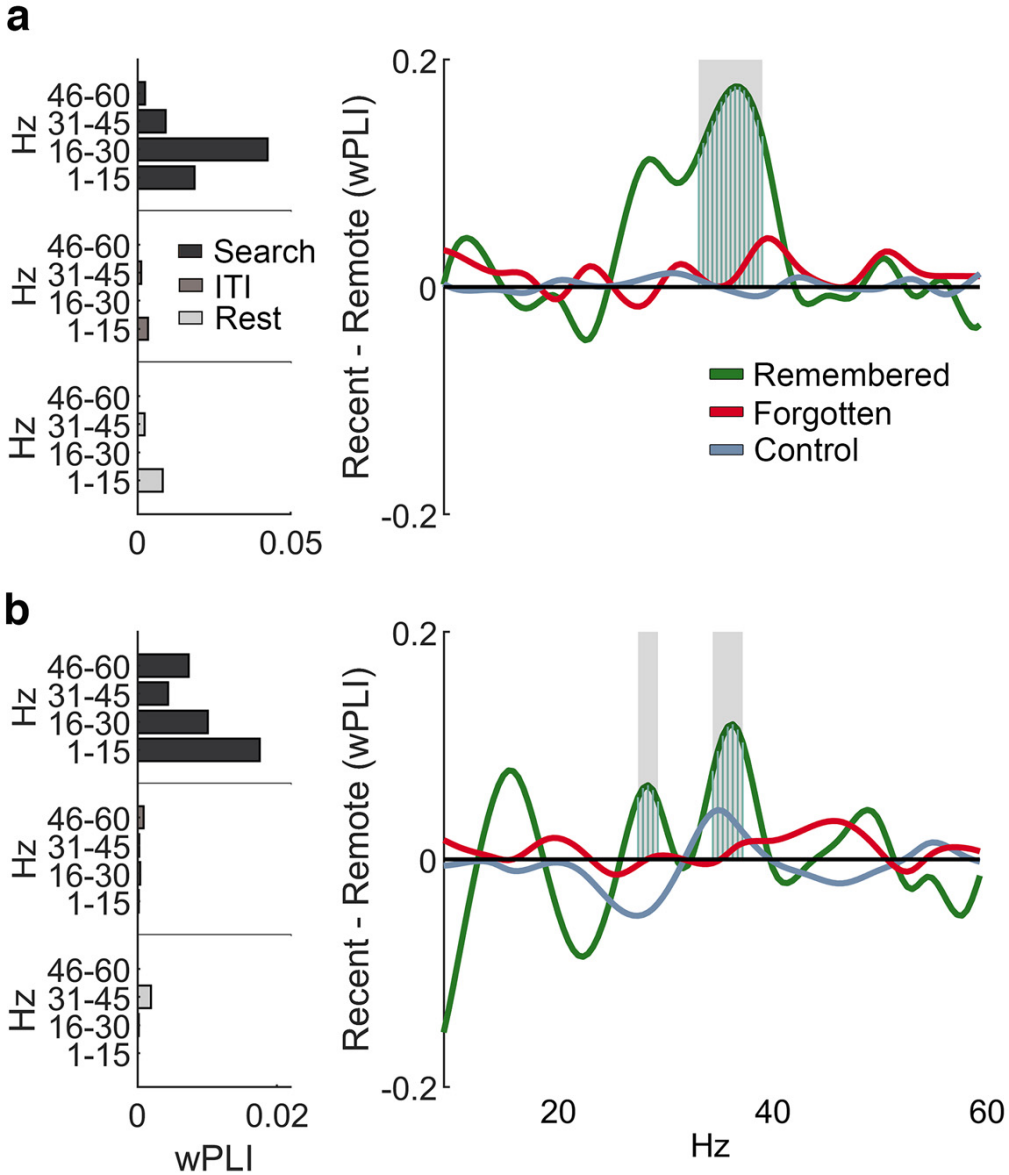
Interarea synchrony between the RSC and the HPC across state and scene memory category. ***a***, Left, Phase-locking (wPLI) by frequency during 10–15 Hz RSC bouts across rest (*n* = 2338), inter-trial interval (ITI) (*n* = 1937), search (*n* = 890) for RI. Right, Difference in RSC–HPC synchrony during alpha bouts in recent and remote trials during remembered, forgotten, and low-alpha control bouts. ***b***, Same as ***a*** but for animal LE; left, rest (*n* = 3346), ITI (*n* = 6423), and search (*n* = 2384); right, remembered (recent, *n* = 112; remote, *n* = 204), forgotten (recent, *n* = 1159; remote, *n* = 909), and control (recent, *n* = 2937; remote, *n* = 2331). Gray shading from zero indicates frequencies where the difference between recent and remote is *p* < 0.05 in a cluster-based permutation test.

**Figure 6.**
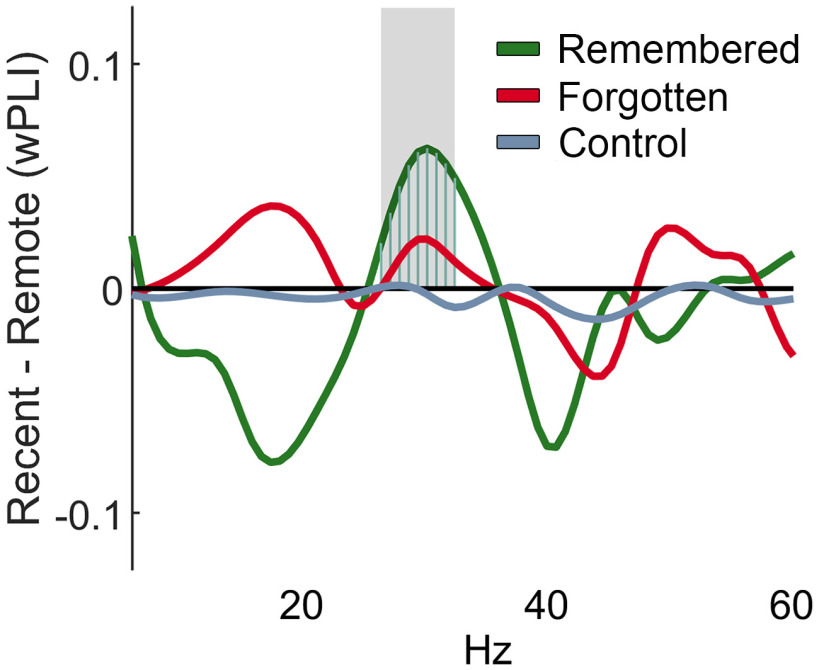
Greater synchrony between ACC and HPC during remembered recent scenes compared with remote scenes. Difference in ACC–HPC phase locking between bouts on recent and remote trials across remembered (recent, 111 bouts; remote, 194 bouts), forgotten trials (recent, 530; remote, 142), and control bouts (recent, 405; remote, 718). Gray shading from zero indicates frequencies with a permutation difference of *p* < 0.05.

In rats and mice, neural ensembles in RSC coordinate their activity with hippocampal ensembles (Destrade and Ott, 1982; Alexander et al., 2018; Mao et al., 2018; Karimi Abadchi et al., 2020; Opalka et al., 2020), and these interactions may be essential for laying down long-lasting mnemonic representations. To assess interareal synchrony between these two structures, we calculated their debiased wPLI during alpha bouts obtained from search, intertrial intervals, and rest epochs. We found that the greatest phase synchrony in both animals occurred during search. We further examined interarea synchrony during successful retrieval, comparing effects for remote and recent retrieval events. We found that during remembered but not forgotten or low-alpha control trials, RSC–HPC synchrony was greater for recent compared with remote scenes, using a cluster-based permutation test corrected for multiple comparisons (*p* < 0.05). The frequencies showing wPLI differences with memory age were observed in the gamma range of ∼25–40 Hz (Fig. 5*a*,*b*; RI recent, remote remembered, *n* = 106, 76; forgotten, *n* = 468, 140; low-alpha control, *n* = 1108, 371; LE recent, remote remembered, *n* = 112, 204; forgotten, *n* = 1159, 909; control *n* = 2937, 2331). Similarly, during the beta-bouts characteristic in the ACC, ACC–HPC synchrony for recent trials was stronger than for remote trials, also in the ∼30 Hz range (Fig. 6).

## Discussion

In this study, we measured neural population activity in the hippocampus, retrosplenial cortex, and anterior cingulate cortex simultaneously as macaques completed an object-scene association task with year-old and newly acquired memoranda. Our results showed that (1) whereas hippocampal activity was invariant across memory age, the retrosplenial cortex responded to old items with greater magnitude alpha (10–15 Hz) and anterior cingulate with greater beta2/gamma (28–35 Hz) oscillations; (2) like the hippocampus, the retrosplenial cortex phase locks to eye movements, but only in the RSC was this locking stronger during retrieval of old items; and (3) during retrieval of newly learned events, gamma-band synchrony between the hippocampus and the two neocortical areas was greater than during retrieval of old events. Few studies have measured RSC activity with direct recordings in primates (Liu et al., 2021), and none to our knowledge during memory guidance or visual search; thus each of these results carry with them a degree of novelty. That said, our results are in general agreement with a role for the retrosplenial cortex in remote memory retrieval and suggest that shifting hippocampal–retrosplenial oscillatory dynamics characterize event retrieval as memories age.

The retrosplenial cortex and broader posterior cingulum in humans is involved in the processing of old episodic or autobiographical memories as well as visuospatial memory and navigation of familiar and virtual environments (Valenstein et al., 1987; Fletcher et al., 1995; Maguire, 2001; Spreng et al., 2009; Vann et al., 2009; Chrastil, 2018). Macaques too rely on the retrosplenial cortex for old episodic-like object-in-scene memory retrieval (Buckley and Mitchell, 2016), which resembles the dependence of such memories on extended hippocampal structures (Gaffan, 1994; Murray et al., 1998; Hampton et al., 2004; Froudist-Walsh et al., 2018; but see Basile and Hampton, 2019). Together, the literature supports a role for the retrosplenial cortex in memory for remote and episodic-like visuospatial events in human and nonhuman primates.

Studies in rats and mice will bear differences in the quality, quantity, and durability of memoranda, corresponding to differences in neural, morphologic, and behavioral specializations across clades. Despite these differences, both the hippocampus and retrosplenial cortex remain sites implicated in spatial/contextual memory retrieval, with an emphasis for a role for RSC in enduring, remote memory retrieval (Corcoran et al., 2011; Tayler et al., 2013; Corcoran et al., 2016; Todd et al., 2018; de Sousa et al., 2019; Trask et al., 2021a). Furthermore, the role of RSC in rodents is not limited to navigation and immersive spatial memory but is also for remote retrieval of items/sensory cue associations (Corcoran et al., 2011; Miller et al., 2014; Todd et al., 2016, 2018; Katche and Medina, 2017; de Landeta et al., 2020; Trask et al., 2021b). We might therefore predict conservation of function for visuospatial object-in-scene task in primates. But what neural dynamics should underlie remote memory retrieval, and how should they change from recent memory retrieval?

We observed a pervasive 10–15 Hz alpha oscillation in this study, which was somewhat unexpected when considered from the well-established vantage point of rat and mouse hippocampal physiology. In rodents (Vann et al., 2009) and rabbits (Talk et al., 2004), the RSC shows theta-band modulation with peaks at 6–8 Hz which appears to interact with hippocampal theta oscillations (Destrade and Ott, 1982; Koike et al., 2017; Alexander et al., 2018) that dominate the spectrum during attentive, locomotor, and active-sensing states (Buzsáki, 1989; Colgin, 2013; Alexander et al., 2020; Nuñez and Buño, 2021). Limited evidence from humans doesn't appear to support equivalent theta oscillations in RSC. In iEEG recordings near RSC, theta power appears to decrease during episodic recall (Foster et al., 2012), although phase locking between the medial temporal lobe and RSC sites was observed during episodic and self-referential tasks in a 3–4 Hz frequency range, with evidence for increases in high, broadband gamma power at each recording site during retrieval (Foster et al., 2013; Dastjerdi et al., 2011). This may simply reflect the need for more studies, or the relative immobility of the participants; however, wireless direct recordings in macaques revealed a decrease in 5–10 Hz theta band in retrosplenial and posterior cingulate cortex during active waking and locomotor behaviors relative to sleep (Talakoub et al., 2019; Figure 1*c*). In contrast, higher frequencies including the 10–20 Hz band increased with alert behaviors. Strikingly, these results were not limited to the RSC in primates; hippocampal theta also appears to be altered. Theta (5–10 Hz) was diminished during waking and walking behaviors, and in midhippocampal and posterior hippocampal sites, alpha bands increased during walking and active states. A closer inspection of the results from other primate studies reveals similar oscillations that are sometimes grouped with slower oscillations. The hippocampal theta literature in humans and monkeys (2–14 Hz) is highly variable in electrode type (micro or macro) and neural signal type (power, phase locking, spike-field coherence), but the fast theta component includes results that overlap those reported here. Specifically, recordings in human and nonhuman primate hippocampus includes >9 Hz alpha-band power (Griffiths et al., 2018; Goyal et al., 2020; Herweg et al., 2020), which has been predictive of impending retrieval (Lega et al., 2012), subsequent memory (Jutras et al., 2013), walking (Bohbot et al., 2017), and wakefulness (Kleen et al., 2021). In contrast, when it has been measured across states, the 6–8 Hz theta power is associated with forgetting (Lega et al., 2012), reflects anesthetized states (Kleen et al., 2021; in macaque, Stewart and Fox, 1991) and rest states (Leonard et al., 2015), or early/slow wave (but not REM) sleep (Uchida et al., 2001; Tamura et al., 2013; Takeuchi et al., 2015; but see Cantero et al., 2003). To summarize, >9 Hz oscillations are seen in the hippocampus and retrosplenial cortex of humans and monkeys, and they have been associated with alert behaviors or more so than sleep-related activity falling in the classic type 1 theta band (6–8 Hz); in the present study we report a stronger alpha modulation in the RSC than in the HPC. To some degree, neural specializations may account for the apparent difference across taxonomic order.

Oscillations at 8–15 Hz are also dominant in posterior medial, parietal, and adjacent occipital regions during visuospatial learning and attention in foveal animals (Lin et al., 2015; Lobier et al., 2018; Foster and Awh, 2019; Jensen et al., 2021), apparently driven by pulvinar inputs (Saalmann et al., 2012; Zhou et al., 2016; Fiebelkorn et al., 2019). The primacy of vision in anthropoid species, and specifically the encoding of visuospatial metrics within structures supporting spatial exploration and memory (Meister and Buffalo, 2016; Rolls and Wirth, 2018; Ryan et al., 2020), may result in a greater influence of visual cortex and thalamus on these structures. This may, in turn, be integrated with a phylogenetically conserved role for the retrosplenial cortex in the gradual development of representations over time (Smith et al., 2012; Makino and Komiyama, 2015; Miller et al., 2019). The connectivity of retrosplenial cortex in macaques situates it as proximate to other regions that are critical for visuospatial processing and for spatiotemporal event memory, consistent with such integration (Parvizi et al., 2006; Vann et al., 2009; Bubb et al., 2017). With few direct studies of retrosplenial cortex physiology in primates, this idea is largely untested, but an intriguing finding in the cat showed that coherent alpha is seen across occipital, hippocampal, and thalamic sites with visual stimulation (Schürmann et al., 2000). Furthermore, alpha-band phase in human posterior/occipital iEEG and posterior-medial MEG was locked to saccades during the encoding of visual stimuli that were later remembered (Staudigl et al., 2017). This, together with our saccade phase-locked results, suggest a coupling between visual search processes, retrosplenial alpha oscillations, and memory retrieval.

An important caution is that field-recorded oscillations are highly degenerate; merely sharing a frequency band does not imply common underlying mechanisms or function. Further work is required to disentangle the circuit generators of these oscillations and the cognitive/behavioral consequences before it will be clear which oscillatory phenomena are common and which are independent across the hippocampus, retrosplenial cortex, and areas related to visual attention.

Despite the common spectral profile we found in retrosplenial and hippocampal regions, their activity differed as a function of memory age. Hippocampal alpha oscillations were unchanging, whereas retrosplenial alpha increased with age, possibly contrasting a constant hippocampal contribution with an emergent or optimized contribution from the retrosplenial cortex. Importantly, the oscillation was present across time, leading to the intriguing finding that within alpha bouts, hippocampal–retrosplenial gamma synchrony was greater during new than old successful memory retrieval. Among various possible accounts, this could indicate the necessity to coordinate (via gamma oscillations) a common but distributed representation across regions during the formative window of new learning, which becomes less critical over time. Hippocampal gamma oscillations are seen during memory formation through excitatory-inhibitory plasticity in parvalbumin-positive neurons seen during learning (He et al., 2021) and lead to network plasticity including increased sharp-wave ripples (SWRs; Zarnadze et al., 2016). Because SWRs are coordinated between the hippocampus and RSC (Alexander et al., 2018), and SWR increases and slow gamma oscillations are associated with event-memory retrieval in macaques (Leonard and Hoffman, 2017; Montefusco-Siegmund et al., 2017; Hussin et al., 2020), the gamma coherence across regions in the present study may serve to facilitate or at least indicate underlying plasticity during early memory formation. We note, however, that decreased HPC–RSC synchrony over time is not necessarily an indication of diminished activity or plasticity within each area; it may signal that the representations within a region have drifted independently, leading to dissimilar recruitment (and presumably resulting in less gamma coupling) during retrieval bouts for old memories (Barry and Maguire, 2019). For a proper understanding of the significance of the nested gamma synchrony that diminishes with memory age, the specific underlying memory representations should be tracked and linked to retrieval. The implication is that these dynamics may contribute to the gradual development of retrosplenial memory representations over time in a way that predicts memory retention (Milczarek et al., 2018) and suggests that cooperative action in hippocampal and retrosplenial populations during and shortly after learning may be important for durable representations (Mao et al., 2018; de Sousa et al., 2019; Milczarek and Vann, 2020).

Considering the paucity of neural data from retrieval of old memories in primates, and specifically from direct measurements of retrosplenial cortex, the present findings offer a strong basis to direct future inquiries into these specific oscillatory phenomena. Furthermore, these oscillations suggest restricted functional windows for long-range coordination during retrieval, which may be region specific and could facilitate the tracking of underlying memory representations over time.
